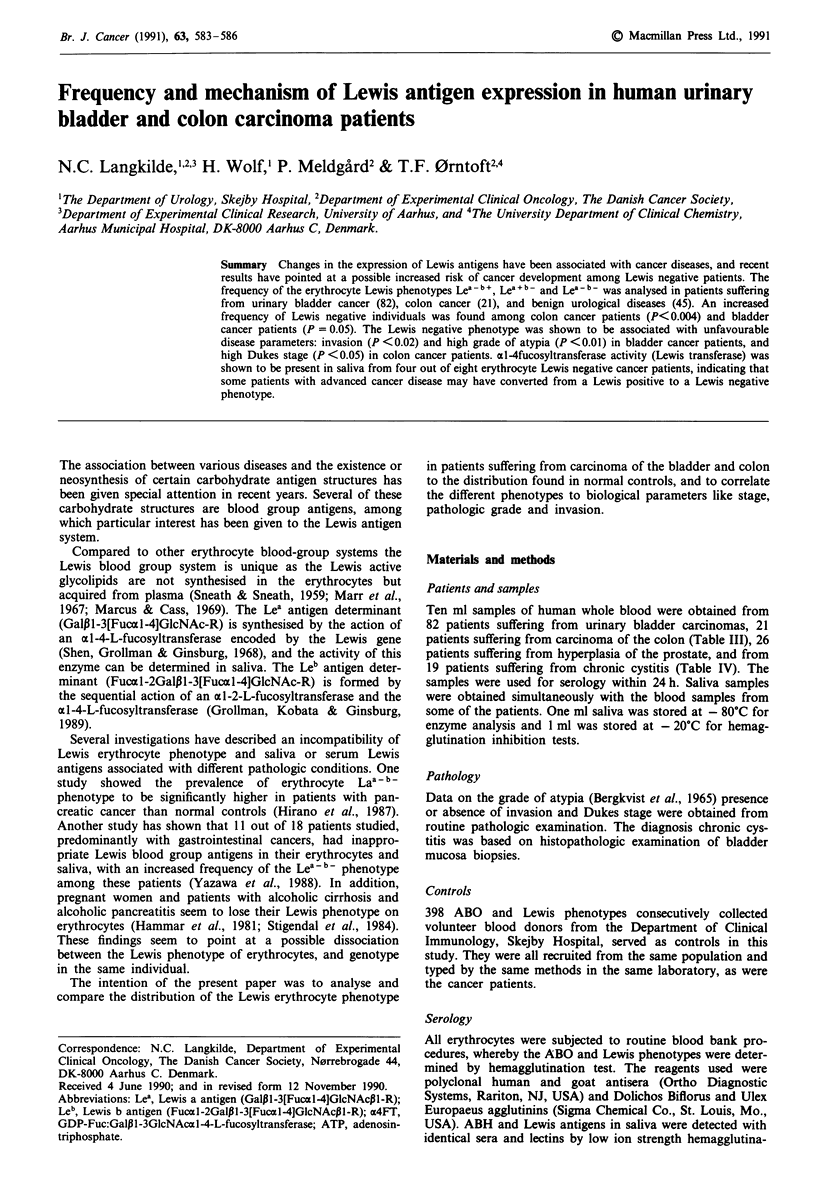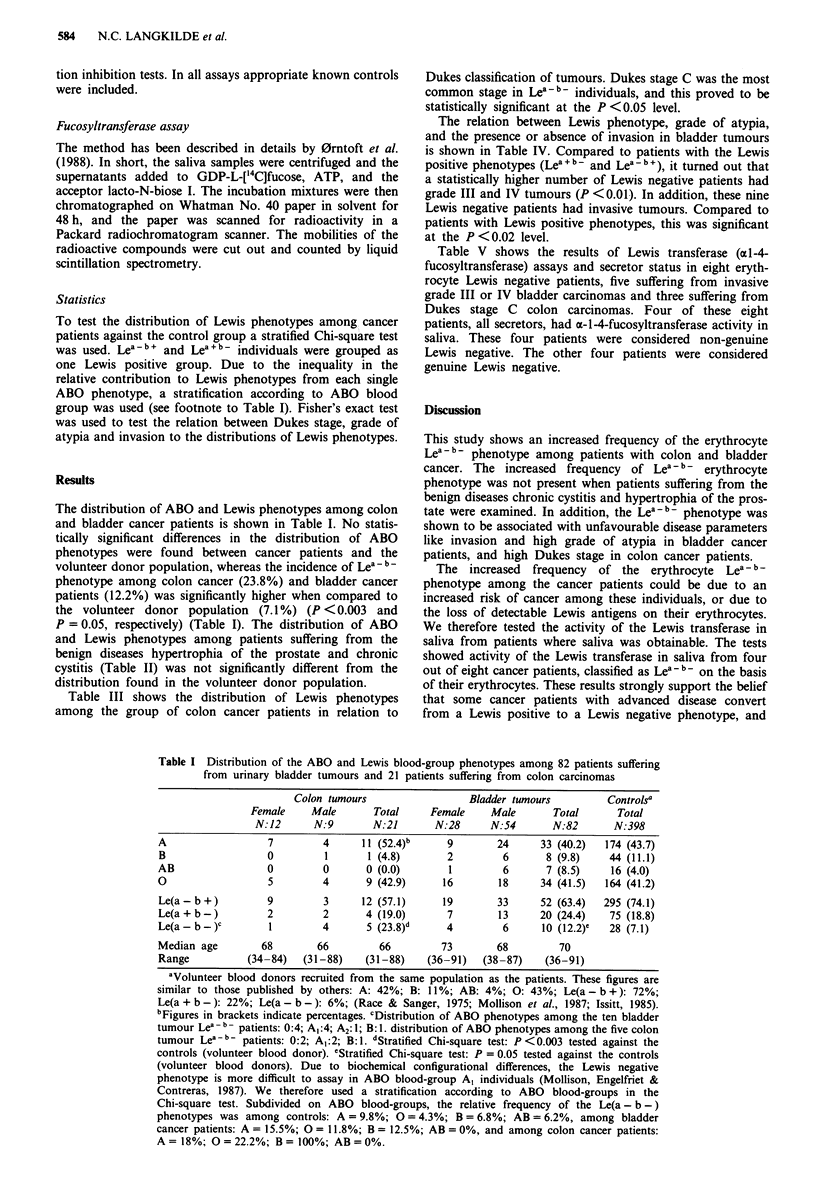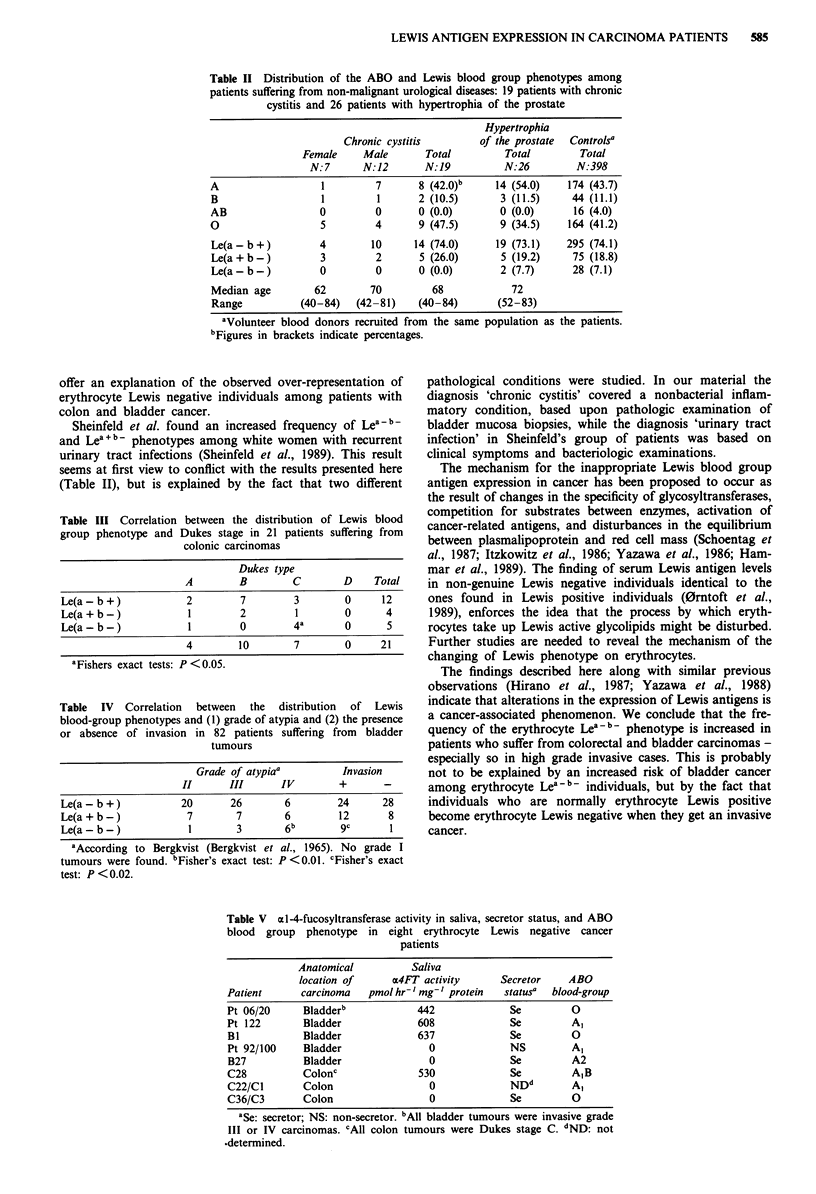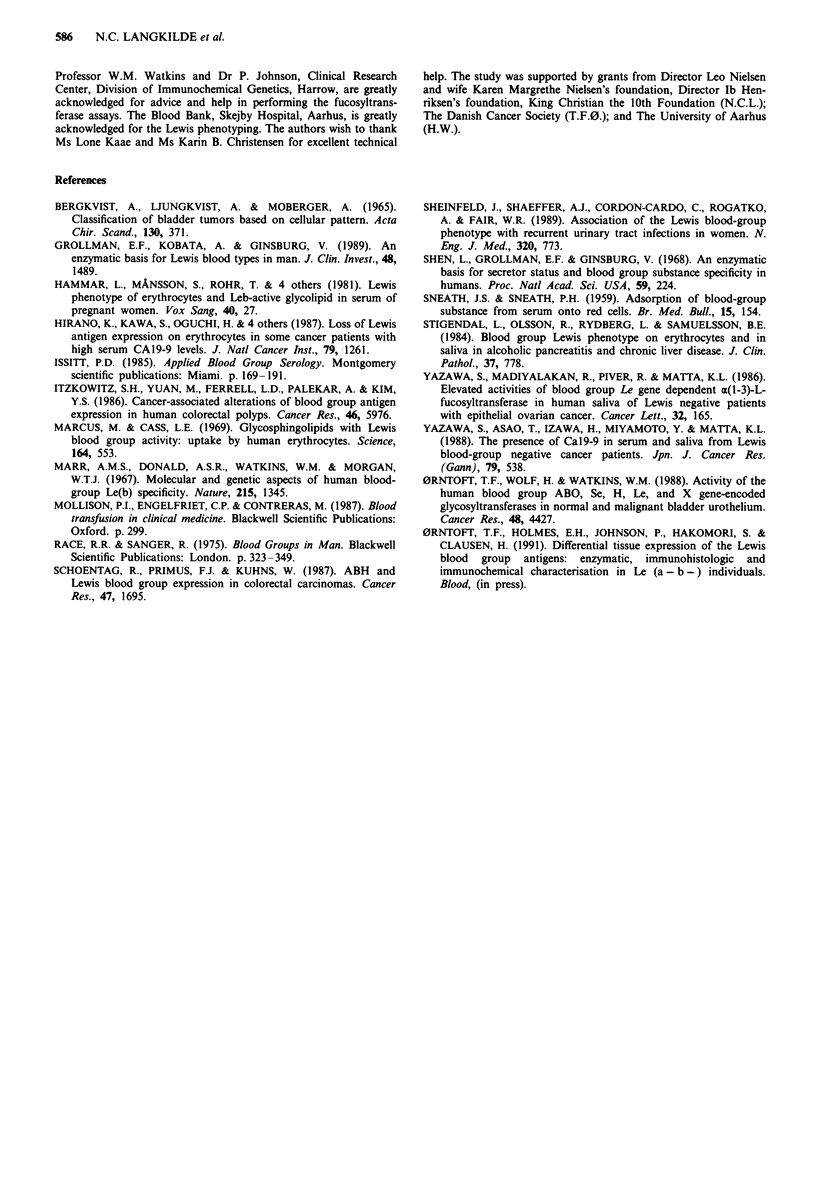# Frequency and mechanism of Lewis antigen expression in human urinary bladder and colon carcinoma patients.

**DOI:** 10.1038/bjc.1991.135

**Published:** 1991-04

**Authors:** N. C. Langkilde, H. Wolf, P. Meldgård, T. F. Orntoft

**Affiliations:** Department of Urology, Skejby Hospital, Denmark.

## Abstract

Changes in the expression of Lewis antigens have been associated with cancer diseases, and recent results have pointed at a possible increased risk of cancer development among Lewis negative patients. The frequency of the erythrocyte Lewis phenotypes Le(a- b+), Le(a+ b-) and Le(a- b-) was analysed in patients suffering from urinary bladder cancer (82), colon cancer (21), and benign urological diseases (45). An increased frequency of Lewis negative individuals was found among colon cancer patients (P less than 0.004) and bladder cancer patients (P = 0.05). The Lewis negative phenotype was shown to be associated with unfavourable disease parameters: invasion (P less than 0.02) and high grade of atypia (P less than 0.01) in bladder cancer patients, and high Dukes stage (P less than 0.05) in colon cancer patients. alpha 1-4fucosyltransferase activity (Lewis transferase) was shown to be present in saliva from four out of eight erythrocyte Lewis negative cancer patients, indicating that some patients with advanced cancer disease may have converted from a Lewis positive to a Lewis negative phenotype.


Br. J. Cancer (1991), 63, 583-586 © Macmillan Press Ltd., 1991

### Frequency and mechanism of Lewis antigen expression in human urinary bladder and colon carcinoma patients

N.C. Langkilde,[1,2,3] H. Wolf,[1] P. Meldgård[2] & T.F. Ørntoft[2,4]

[1]*The Department of Urology, Skejby Hospital,* [2]*Department of Experimental Clinical Oncology, The Danish Cancer Society,* [3]*Department of Experimental Clinical Research, University of Aarhus, and* [4]*The University Department of Clinical Chemistry, Aarhus Municipal Hospital, DK-8000 Aarhus C, Denmark.*

**Summary** Changes in the expression of Lewis antigens have been associated with cancer diseases, and recent results have pointed at a possible increased risk of cancer development among Lewis negative patients. The frequency of the erythrocyte Lewis phenotypes $Le^{a-b+}$, $Le^{a+b-}$ and $Le^{a-b-}$ was analysed in patients suffering from urinary bladder cancer (82), colon cancer (21), and benign urological diseases (45). An increased frequency of Lewis negative individuals was found among colon cancer patients ($P<0.004$) and bladder cancer patients ($P = 0.05$). The Lewis negative phenotype was shown to be associated with unfavourable disease parameters: invasion ($P<0.02$) and high grade of atypia ($P<0.01$) in bladder cancer patients, and high Dukes stage ($P<0.05$) in colon cancer patients. α1-4fucosyltransferase activity (Lewis transferase) was shown to be present in saliva from four out of eight erythrocyte Lewis negative cancer patients, indicating that some patients with advanced cancer disease may have converted from a Lewis positive to a Lewis negative phenotype.

The association between various diseases and the existence or neosynthesis of certain carbohydrate antigen structures has been given special attention in recent years. Several of these carbohydrate structures are blood group antigens, among which particular interest has been given to the Lewis antigen system.

Compared to other erythrocyte blood-group systems the Lewis blood group system is unique as the Lewis active glycolipids are not synthesised in the erythrocytes but acquired from plasma (Sneath & Sneath, 1959; Marr *et al.*, 1967; Marcus & Cass, 1969). The $Le^a$ antigen determinant (Galβ1-3[Fucα1-4]GlcNAc-R) is synthesised by the action of an α1-4-L-fucosyltransferase encoded by the Lewis gene (Shen, Grollman & Ginsburg, 1968), and the activity of this enzyme can be determined in saliva. The $Le^b$ antigen determinant (Fucα1-2Galβ1-3[Fucα1-4]GlcNAc-R) is formed by the sequential action of an α1-2-L-fucosyltransferase and the α1-4-L-fucosyltransferase (Grollman, Kobata & Ginsburg, 1989).

Several investigations have described an incompatibility of Lewis erythrocyte phenotype and saliva or serum Lewis antigens associated with different pathologic conditions. One study showed the prevalence of erythrocyte $La^{a-b-}$ phenotype to be significantly higher in patients with pancreatic cancer than normal controls (Hirano *et al.*, 1987). Another study has shown that 11 out of 18 patients studied, predominantly with gastrointestinal cancers, had inappropriate Lewis blood group antigens in their erythrocytes and saliva, with an increased frequency of the $Le^{a-b-}$ phenotype among these patients (Yazawa *et al.*, 1988). In addition, pregnant women and patients with alcoholic cirrhosis and alcoholic pancreatitis seem to lose their Lewis phenotype on erythrocytes (Hammar *et al.*, 1981; Stigendal *et al.*, 1984). These findings seem to point at a possible dissociation between the Lewis phenotype of erythrocytes, and genotype in the same individual.

The intention of the present paper was to analyse and compare the distribution of the Lewis erythrocyte phenotype in patients suffering from carcinoma of the bladder and colon to the distribution found in normal controls, and to correlate the different phenotypes to biological parameters like stage, pathologic grade and invasion.

### Materials and methods

### *Patients and samples*

Ten ml samples of human whole blood were obtained from 82 patients suffering from urinary bladder carcinomas, 21 patients suffering from carcinoma of the colon (Table III), 26 patients suffering from hyperplasia of the prostate, and from 19 patients suffering from chronic cystitis (Table IV). The samples were used for serology within 24 h. Saliva samples were obtained simultaneously with the blood samples from some of the patients. One ml saliva was stored at − 80°C for enzyme analysis and 1 ml was stored at − 20°C for hemagglutination inhibition tests.

### *Pathology*

Data on the grade of atypia (Bergkvist *et al.*, 1965) presence or absence of invasion and Dukes stage were obtained from routine pathologic examination. The diagnosis chronic cystitis was based on histopathologic examination of bladder mucosa biopsies.

### *Controls*

398 ABO and Lewis phenotypes consecutively collected volunteer blood donors from the Department of Clinical Immunology, Skejby Hospital, served as controls in this study. They were all recruited from the same population and typed by the same methods in the same laboratory, as were the cancer patients.

### *Serology*

All erythrocytes were subjected to routine blood bank procedures, whereby the ABO and Lewis phenotypes were determined by hemagglutination test. The reagents used were polyclonal human and goat antisera (Ortho Diagnostic Systems, Rariton, NJ, USA) and Dolichos Biflorus and Ulex Europaeus agglutinins (Sigma Chemical Co., St. Louis, Mo., USA). ABH and Lewis antigens in saliva were detected with identical sera and lectins by low ion strength hemagglutina-

Correspondence: N.C. Langkilde, Department of Experimental Clinical Oncology, The Danish Cancer Society, Nørrebrogade 44, DK-8000 Aarhus C. Denmark.

Received 4 June 1990; and in revised form 12 November 1990.

Abbreviations: $Le^a$, Lewis a antigen (Galβ1-3[Fucα1-4]GlcNAcβ1-R); $Le^b$, Lewis b antigen (Fucα1-2Galβ1-3[Fucα1-4]GlcNAcβ1-R); α4FT, GDP-Fuc:Galβ1-3GlcNAcα1-4-L-fucosyltransferase; ATP, adenosintriphosphate.

tion inhibition tests. In all assays appropriate known controls were included.

*Fucosyltransferase assay*

The method has been described in details by Ørntoft *et al.* (1988). In short, the saliva samples were centrifuged and the supernatants added to GDP-L-[$^{14}$C]fucose, ATP, and the acceptor lacto-N-biose I. The incubation mixtures were then chromatographed on Whatman No. 40 paper in solvent for 48 h, and the paper was scanned for radioactivity in a Packard radiochromatogram scanner. The mobilities of the radioactive compounds were cut out and counted by liquid scintillation spectrometry.

*Statistics*

To test the distribution of Lewis phenotypes among cancer patients against the control group a stratified Chi-square test was used. $Le^{a-b+}$ and $Le^{a+b-}$ individuals were grouped as one Lewis positive group. Due to the inequality in the relative contribution to Lewis phenotypes from each single ABO phenotype, a stratification according to ABO blood group was used (see footnote to Table I). Fisher's exact test was used to test the relation between Dukes stage, grade of atypia and invasion to the distributions of Lewis phenotypes.

### Results

The distribution of ABO and Lewis phenotypes among colon and bladder cancer patients is shown in Table I. No statistically significant differences in the distribution of ABO phenotypes were found between cancer patients and the volunteer donor population, whereas the incidence of $Le^{a-b-}$ phenotype among colon cancer (23.8%) and bladder cancer patients (12.2%) was significantly higher when compared to the volunteer donor population (7.1%) ($P < 0.003$ and $P = 0.05$, respectively) (Table I). The distribution of ABO and Lewis phenotypes among patients suffering from the benign diseases hypertrophia of the prostate and chronic cystitis (Table II) was not significantly different from the distribution found in the volunteer donor population.

Table III shows the distribution of Lewis phenotypes among the group of colon cancer patients in relation to Dukes classification of tumours. Dukes stage C was the most common stage in $Le^{a-b-}$ individuals, and this proved to be statistically significant at the $P < 0.05$ level.

The relation between Lewis phenotype, grade of atypia, and the presence or absence of invasion in bladder tumours is shown in Table IV. Compared to patients with the Lewis positive phenotypes ($Le^{a+b-}$ and $Le^{a-b+}$), it turned out that a statistically higher number of Lewis negative patients had grade III and IV tumours ($P < 0.01$). In addition, these nine Lewis negative patients had invasive tumours. Compared to patients with Lewis positive phenotypes, this was significant at the $P < 0.02$ level.

Table V shows the results of Lewis transferase (α1-4-fucosyltransferase) assays and secretor status in eight erythrocyte Lewis negative patients, five suffering from invasive grade III or IV bladder carcinomas and three suffering from Dukes stage C colon carcinomas. Four of these eight patients, all secretors, had α-1-4-fucosyltransferase activity in saliva. These four patients were considered non-genuine Lewis negative. The other four patients were considered genuine Lewis negative.

### Discussion

This study shows an increased frequency of the erythrocyte $Le^{a-b-}$ phenotype among patients with colon and bladder cancer. The increased frequency of $Le^{a-b-}$ erythrocyte phenotype was not present when patients suffering from the benign diseases chronic cystitis and hypertrophia of the prostate were examined. In addition, the $Le^{a-b-}$ phenotype was shown to be associated with unfavourable disease parameters like invasion and high grade of atypia in bladder cancer patients, and high Dukes stage in colon cancer patients.

The increased frequency of the erythrocyte $Le^{a-b-}$ phenotype among the cancer patients could be due to an increased risk of cancer among these individuals, or due to the loss of detectable Lewis antigens on their erythrocytes. We therefore tested the activity of the Lewis transferase in saliva from patients where saliva was obtainable. The tests showed activity of the Lewis transferase in saliva from four out of eight cancer patients, classified as $Le^{a-b-}$ on the basis of their erythrocytes. These results strongly support the belief that some cancer patients with advanced disease convert from a Lewis positive to a Lewis negative phenotype, and

**Table I** Distribution of the ABO and Lewis blood-group phenotypes among 82 patients suffering from urinary bladder tumours and 21 patients suffering from colon carcinomas

| | *Colon tumours* | | | *Bladder tumours* | | | *Controls*[a] |
|---|---|---|---|---|---|---|---|
| | *Female* *N:12* | *Male* *N:9* | *Total* *N:21* | *Female* *N:28* | *Male* *N:54* | *Total* *N:82* | *Total* *N:398* |
| A | 7 | 4 | 11 (52.4)[b] | 9 | 24 | 33 (40.2) | 174 (43.7) |
| B | 0 | 1 | 1 (4.8) | 2 | 6 | 8 (9.8) | 44 (11.1) |
| AB | 0 | 0 | 0 (0.0) | 1 | 6 | 7 (8.5) | 16 (4.0) |
| O | 5 | 4 | 9 (42.9) | 16 | 18 | 34 (41.5) | 164 (41.2) |
| Le(a − b +) | 9 | 3 | 12 (57.1) | 19 | 33 | 52 (63.4) | 295 (74.1) |
| Le(a + b −) | 2 | 2 | 4 (19.0) | 7 | 13 | 20 (24.4) | 75 (18.8) |
| Le(a − b −)[c] | 1 | 4 | 5 (23.8)[d] | 4 | 6 | 10 (12.2)[e] | 28 (7.1) |
| Median age | 68 | 66 | 66 | 73 | 68 | 70 | |
| Range | (34–84) | (31–88) | (31–88) | (36–91) | (38–87) | (36–91) | |

[a]Volunteer blood donors recruited from the same population as the patients. These figures are similar to those published by others: A: 42%; B: 11%; AB: 4%; O: 43%; Le(a − b +): 72%; Le(a + b −): 22%; Le(a − b −): 6%; (Race & Sanger, 1975; Mollison *et al.*, 1987; Issitt, 1985). [b]Figures in brackets indicate percentages. [c]Distribution of ABO phenotypes among the ten bladder tumour $Le^{a-b-}$ patients: 0:4; $A_1$:4; $A_2$:1; B:1. distribution of ABO phenotypes among the five colon tumour $Le^{a-b-}$ patients: 0:2; $A_1$:2; B:1. [d]Stratified Chi-square test: $P < 0.003$ tested against the controls (volunteer blood donor). [e]Stratified Chi-square test: $P = 0.05$ tested against the controls (volunteer blood donors). Due to biochemical configurational differences, the Lewis negative phenotype is more difficult to assay in ABO blood-group $A_1$ individuals (Mollison, Engelfriet & Contreras, 1987). We therefore used a stratification according to ABO blood-groups in the Chi-square test. Subdivided on ABO blood-groups, the relative frequency of the Le(a − b −) phenotypes was among controls: A = 9.8%; O = 4.3%; B = 6.8%; AB = 6.2%, among bladder cancer patients: A = 15.5%; O = 11.8%; B = 12.5%; AB = 0%, and among colon cancer patients: A = 18%; O = 22.2%; B = 100%; AB = 0%.

**Table II** Distribution of the ABO and Lewis blood group phenotypes among patients suffering from non-malignant urological diseases: 19 patients with chronic cystitis and 26 patients with hypertrophia of the prostate

| | *Chronic cystitis* Female N:7 | Male N:12 | Total N:19 | *Hypertrophia of the prostate* Total N:26 | *Controls*[a] Total N:398 |
|---|---|---|---|---|---|
| A | 1 | 7 | 8 (42.0)[b] | 14 (54.0) | 174 (43.7) |
| B | 1 | 1 | 2 (10.5) | 3 (11.5) | 44 (11.1) |
| AB | 0 | 0 | 0 (0.0) | 0 (0.0) | 16 (4.0) |
| O | 5 | 4 | 9 (47.5) | 9 (34.5) | 164 (41.2) |
| Le(a − b + ) | 4 | 10 | 14 (74.0) | 19 (73.1) | 295 (74.1) |
| Le(a + b − ) | 3 | 2 | 5 (26.0) | 5 (19.2) | 75 (18.8) |
| Le(a − b − ) | 0 | 0 | 0 (0.0) | 2 (7.7) | 28 (7.1) |
| Median age | 62 | 70 | 68 | 72 | |
| Range | (40–84) | (42–81) | (40–84) | (52–83) | |

[a]Volunteer blood donors recruited from the same population as the patients. [b]Figures in brackets indicate percentages.

offer an explanation of the observed over-representation of erythrocyte Lewis negative individuals among patients with colon and bladder cancer.

Sheinfeld *et al.* found an increased frequency of $Le^{a-b-}$ and $Le^{a+b-}$ phenotypes among white women with recurrent urinary tract infections (Sheinfeld *et al.*, 1989). This result seems at first view to conflict with the results presented here (Table II), but is explained by the fact that two different pathological conditions were studied. In our material the diagnosis 'chronic cystitis' covered a nonbacterial inflammatory condition, based upon pathologic examination of bladder mucosa biopsies, while the diagnosis 'urinary tract infection' in Sheinfeld's group of patients was based on clinical symptoms and bacteriologic examinations.

**Table III** Correlation between the distribution of Lewis blood group phenotype and Dukes stage in 21 patients suffering from colonic carcinomas

| | *Dukes type* A | B | C | D | Total |
|---|---|---|---|---|---|
| Le(a − b + ) | 2 | 7 | 3 | 0 | 12 |
| Le(a + b − ) | 1 | 2 | 1 | 0 | 4 |
| Le(a − b − ) | 1 | 0 | 4[a] | 0 | 5 |
| | 4 | 10 | 7 | 0 | 21 |

[a]Fishers exact tests: $P < 0.05$.

**Table IV** Correlation between the distribution of Lewis blood-group phenotypes and (1) grade of atypia and (2) the presence or absence of invasion in 82 patients suffering from bladder tumours

| | *Grade of atypia*[a] II | III | IV | *Invasion* + | − |
|---|---|---|---|---|---|
| Le(a − b + ) | 20 | 26 | 6 | 24 | 28 |
| Le(a + b − ) | 7 | 7 | 6 | 12 | 8 |
| Le(a − b − ) | 1 | 3 | 6[b] | 9[c] | 1 |

[a]According to Bergkvist (Bergkvist *et al.*, 1965). No grade I tumours were found. [b]Fisher's exact test: $P < 0.01$. [c]Fisher's exact test: $P < 0.02$.

The mechanism for the inappropriate Lewis blood group antigen expression in cancer has been proposed to occur as the result of changes in the specificity of glycosyltransferases, competition for substrates between enzymes, activation of cancer-related antigens, and disturbances in the equilibrium between plasmalipoprotein and red cell mass (Schoentag *et al.*, 1987; Itzkowitz *et al.*, 1986; Yazawa *et al.*, 1986; Hammar *et al.*, 1989). The finding of serum Lewis antigen levels in non-genuine Lewis negative individuals identical to the ones found in Lewis positive individuals (Ørntoft *et al.*, 1989), enforces the idea that the process by which erythrocytes take up Lewis active glycolipids might be disturbed. Further studies are needed to reveal the mechanism of the changing of Lewis phenotype on erythrocytes.

The findings described here along with similar previous observations (Hirano *et al.*, 1987; Yazawa *et al.*, 1988) indicate that alterations in the expression of Lewis antigens is a cancer-associated phenomenon. We conclude that the frequency of the erythrocyte $Le^{a-b-}$ phenotype is increased in patients who suffer from colorectal and bladder carcinomas – especially so in high grade invasive cases. This is probably not to be explained by an increased risk of bladder cancer among erythrocyte $Le^{a-b-}$ individuals, but by the fact that individuals who are normally erythrocyte Lewis positive become erythrocyte Lewis negative when they get an invasive cancer.

**Table V** α1-4-fucosyltransferase activity in saliva, secretor status, and ABO blood group phenotype in eight erythrocyte Lewis negative cancer patients

| *Patient* | *Anatomical location of carcinoma* | *Saliva α4FT activity pmol hr⁻¹ mg⁻¹ protein* | *Secretor status*[a] | *ABO blood-group* |
|---|---|---|---|---|
| Pt 06/20 | Bladder[b] | 442 | Se | O |
| Pt 122 | Bladder | 608 | Se | $A_1$ |
| B1 | Bladder | 637 | Se | O |
| Pt 92/100 | Bladder | 0 | NS | $A_1$ |
| B27 | Bladder | 0 | Se | A2 |
| C28 | Colon[c] | 530 | Se | $A_1B$ |
| C22/C1 | Colon | 0 | ND[d] | $A_1$ |
| C36/C3 | Colon | 0 | Se | O |

[a]Se: secretor; NS: non-secretor. [b]All bladder tumours were invasive grade III or IV carcinomas. [c]All colon tumours were Dukes stage C. [d]ND: not determined.

Professor W.M. Watkins and Dr P. Johnson, Clinical Research Center, Division of Immunochemical Genetics, Harrow, are greatly acknowledged for advice and help in performing the fucosyltransferase assays. The Blood Bank, Skejby Hospital, Aarhus, is greatly acknowledged for the Lewis phenotyping. The authors wish to thank Ms Lone Kaae and Ms Karin B. Christensen for excellent technical help. The study was supported by grants from Director Leo Nielsen and wife Karen Margrethe Nielsen's foundation, Director Ib Henriksen's foundation, King Christian the 10th Foundation (N.C.L.); The Danish Cancer Society (T.F.Ø.); and The University of Aarhus (H.W.).

### References

BERGKVIST, A., LJUNGKVIST, A. & MOBERGER, A. (1965). Classification of bladder tumors based on cellular pattern. *Acta Chir. Scand.*, **130**, 371.

GROLLMAN, E.F., KOBATA, A. & GINSBURG, V. (1989). An enzymatic basis for Lewis blood types in man. *J. Clin. Invest.*, **48**, 1489.

HAMMAR, L., MÅNSSON, S., ROHR, T. & 4 others (1981). Lewis phenotype of erythrocytes and Leb-active glycolipid in serum of pregnant women. *Vox Sang*, **40**, 27.

HIRANO, K., KAWA, S., OGUCHI, H. & 4 others (1987). Loss of Lewis antigen expression on erythrocytes in some cancer patients with high serum CA19-9 levels. *J. Natl Cancer Inst.*, **79**, 1261.

ISSITT, P.D. (1985). *Applied Blood Group Serology*. Montgomery scientific publications: Miami. p. 169–191.

ITZKOWITZ, S.H., YUAN, M., FERRELL, L.D., PALEKAR, A. & KIM, Y.S. (1986). Cancer-associated alterations of blood group antigen expression in human colorectal polyps. *Cancer Res.*, **46**, 5976.

MARCUS, M. & CASS, L.E. (1969). Glycosphingolipids with Lewis blood group activity: uptake by human erythrocytes. *Science*, **164**, 553.

MARR, A.M.S., DONALD, A.S.R., WATKINS, W.M. & MORGAN, W.T.J. (1967). Molecular and genetic aspects of human blood-group Le(b) specificity. *Nature*, **215**, 1345.

MOLLISON, P.I., ENGELFRIET, C.P. & CONTRERAS, M. (1987). *Blood transfusion in clinical medicine*. Blackwell Scientific Publications: Oxford. p. 299.

RACE, R.R. & SANGER, R. (1975). *Blood Groups in Man*. Blackwell Scientific Publications: London. p. 323–349.

SCHOENTAG, R., PRIMUS, F.J. & KUHNS, W. (1987). ABH and Lewis blood group expression in colorectal carcinomas. *Cancer Res.*, **47**, 1695.

SHEINFELD, J., SHAEFFER, A.J., CORDON-CARDO, C., ROGATKO, A. & FAIR, W.R. (1989). Association of the Lewis blood-group phenotype with recurrent urinary tract infections in women. *N. Eng. J. Med.*, **320**, 773.

SHEN, L., GROLLMAN, E.F. & GINSBURG, V. (1968). An enzymatic basis for secretor status and blood group substance specificity in humans. *Proc. Natl Acad. Sci. USA*, **59**, 224.

SNEATH, J.S. & SNEATH, P.H. (1959). Adsorption of blood-group substance from serum onto red cells. *Br. Med. Bull.*, **15**, 154.

STIGENDAL, L., OLSSON, R., RYDBERG, L. & SAMUELSSON, B.E. (1984). Blood group Lewis phenotype on erythrocytes and in saliva in alcoholic pancreatitis and chronic liver disease. *J. Clin. Pathol.*, **37**, 778.

YAZAWA, S., MADIYALAKAN, R., PIVER, R. & MATTA, K.L. (1986). Elevated activities of blood group *Le* gene dependent α(1-3)-L-fucosyltransferase in human saliva of Lewis negative patients with epithelial ovarian cancer. *Cancer Lett.*, **32**, 165.

YAZAWA, S., ASAO, T., IZAWA, H., MIYAMOTO, Y. & MATTA, K.L. (1988). The presence of Ca19-9 in serum and saliva from Lewis blood-group negative cancer patients. *Jpn. J. Cancer Res. (Gann)*, **79**, 538.

ØRNTOFT, T.F., WOLF, H. & WATKINS, W.M. (1988). Activity of the human blood group ABO, Se, H, Le, and X gene-encoded glycosyltransferases in normal and malignant bladder urothelium. *Cancer Res.*, **48**, 4427.

ØRNTOFT, T.F., HOLMES, E.H., JOHNSON, P., HAKOMORI, S. & CLAUSEN, H. (1991). Differential tissue expression of the Lewis blood group antigens: enzymatic, immunohistologic and immunochemical characterisation in Le (a – b –) individuals. *Blood*, (in press).